# Pre-Pregnancy BMI, Gestational Weight Gain, and the Risk of Hypertensive Disorders of Pregnancy: A Cohort Study in Wuhan, China

**DOI:** 10.1371/journal.pone.0136291

**Published:** 2015-08-25

**Authors:** Aifen Zhou, Chao Xiong, Ronghua Hu, Yiming Zhang, Bryan A. Bassig, Elizabeth Triche, Shaoping Yang, Lin Qiu, Yaqi Zhang, Cong Yao, Shunqing Xu, Youjie Wang, Wei Xia, Zhengmin Qian, Tongzhang Zheng, Bin Zhang

**Affiliations:** 1 Wuhan Women and Children Health Care Center, Wuhan, Hubei, People’s Republic of China; 2 Department of Environmental Health Sciences, Yale School of Public Health, New Haven, CT, United States of America; 3 Department of Epidemiology, Brown University School of Public Health, Providence, RI, United States of America; 4 Key Laboratory of Environment and Health, School of Public Health, Tongji Medical College, Huazhong University of Science and Technology, Wuhan, Hubei, People’s Republic of China; 5 Department of Epidemiology, College for Public Health and Social Justice, Saint Louis University, MO, United States of America; University of Missouri, UNITED STATES

## Abstract

**Background:**

Hypertensive disorders of pregnancy (HDP) are major causes of maternal death worldwide and the risk factors are not fully understood. Few studies have investigated the risk factors for HDP among Chinese women. A cohort study involving 84,656 women was conducted to investigate pre-pregnancy BMI, total gestational weight gain (GWG), and GWG during early pregnancy as risk factors for HDP among Chinese women.

**Methods:**

The study was conducted between 2011–2013 in Wuhan, China, utilizing data from the Maternal and Children Healthcare Information Tracking System of Wuhan. A total of 84,656 women with a live singleton pregnancy were included. Multiple unconditional logistic regression was conducted to evaluate associations between putative risk factors and HDP.

**Results:**

Women who were overweight or obese before pregnancy had an elevated risk of developing HDP (overweight: OR = 2.66, 95% CI = 2.32–3.05; obese: OR = 5.53, 95% CI = 4.28–7.13) compared to their normal weight counterparts. Women with total GWG above the Institute of Medicine (IOM) recommendation had an adjusted OR of 1.72 (95% CI = 1.54–1.93) for HDP compared to women who had GWG within the IOM recommendation. Women with gestational BMI gain >10 kg/m^2^ during pregnancy had an adjusted OR of 3.35 (95% CI = 2.89–3.89) for HDP, compared to women with a gestational BMI gain <5 kg/m^2^. The increased risk of HDP was also observed among women with higher early pregnancy (up to 18 weeks of pregnancy) GWG (>600g/wk: adjusted OR = 1.48, 95% CI = 1.19–1.84).

**Conclusion:**

The results from this study show that maternal pre-pregnancy BMI, early GWG, and total GWG are positively associated with the risk of HDP. Weight control efforts before and during pregnancy may help to reduce the risk of HDP.

## Introduction

Hypertensive disorders of pregnancy (HDP), consisting of gestational hypertension (GH) and preeclampsia (PE), are major causes of maternal and perinatal morbidity and mortality [1]. It has been reported that HDP complicates 5–10% of all pregnancies worldwide and causes up to 70,000 maternal deaths each year [2]. In addition to maternal complications, HDP is also associated with fetal intrauterine growth restriction and preterm birth [3]. However, effective treatments for HDP are still limited, and currently the etiology of HDP is not completely understood [4].

Previous studies in developed countries have linked pre-pregnancy obesity to the development of HDP suggesting that the risk factors for this condition may not be limited to exposures during the gestational period [5, 6]. On the other hand, there is increasing concern about whether gestational weight gain (GWG) may influence the development of HDP and to what extent control of GWG can reduce the risk of HDP. Several studies have indicated that HDP is more likely to develop in women with greater GWG [7–9]. However, the majority of previous studies have only evaluated total GWG during pregnancy in relation to risk of HDP; given that women with HDP are likely to experience edema during pregnancy [10], which may result in greater GWG, it is hard to distinguish the weight gain caused by edema from the weight gain independent of this condition [11]. Therefore, whether GWG is causally related to the development of HDP is still unclear. To our knowledge, there has been only one study, which was conducted in the United Kingdom, that addressed this issue by assessing weight gain during early pregnancy that was likely not the result of edema. Some positive associations between greater GWG in early pregnancy and increased risk of developing gestational hypertension and preeclampsia were observed in that study [11].

Although the impact of HDP is thought to be much more severe in developing countries than in developed countries [12], there are limited epidemiological studies that have evaluated risk factors for HDP among women in developing countries, including in China. As Asian women generally have a lower BMI prior to pregnancy compared to women in Western countries [13], the relationship between pre-pregnancy BMI, GWG, and risk of HDP among Asian women may differ, although these hypotheses have not been extensively studied. Therefore, we conducted a cohort study to evaluate the association of pre-pregnancy BMI, total GWG, and early pregnancy GWG with risk of HDP among a relatively large population of women from Wuhan, China.

## Methods

### Study Population

This is a cohort study conducted in Wuhan, China, utilizing data from the Maternal and Children Healthcare Information Tracking System of Wuhan. The tracking system includes information pertaining to demographic characteristics, medical history, prenatal examinations, deliveries, and postnatal visits for mothers and infants from all of the 93 hospitals and 121 community health centers in Wuhan. Enrolled women in our study included those without a history of chronic hypertension or cardiovascular disease prior to pregnancy, who lived in the urban area of Wuhan during pregnancy, and who delivered a live singleton newborn with no birth defects and gestational age no less than 28 weeks between June 1, 2011 and June 10, 2013.

A total of 97,582 women were enrolled initially. We then excluded those with any missing values for height, pre-pregnancy weight, or GWG. To eliminate extreme outliers, data analysis was also limited to women whose height, weight, and GWG were within 5 standard deviations of the mean. A total of 84,656 women met these criteria and were included in the analysis; 63,603 of them had a record of at least one weight measurement during early pregnancy (8–18 weeks). Informed consent was not obtained because data from this study were abstracted from the healthcare information system without individual identification, and the research protocol was approved by the Institutional Review Board of Wuhan Women and Children Health Care Center, in accordance to the principles of the Declaration of Helsinki. All patient records were anonymized and de-identified prior to analysis.

### Exposure Information

Weight measurements during pregnancy were taken routinely as part of antenatal care at the clinic. Pre-pregnancy weight and height were self-reported at the first antenatal care visit (usually in the first trimester). Pre-pregnancy BMI was calculated as weight (in kg)/height (in meters) squared and categorized into four groups based on recommendations by the Working Group On Obesity in China of the Chinese Ministry of Health: underweight (<18.5 kg/m^2^), normal weight (18.5–23.9 kg/m^2^), overweight (24–27.9 kg/m^2^), and obese (≥28 kg/m^2^) [14].

Total GWG was calculated by subtracting pre-pregnancy weight from the weight on delivery day. GWG was categorized according to the recommendations of the Institute of Medicine (IOM) (2009) [15]. GWG within the IOM recommendations was defined as 12.5–18 kg, 11.5–16 kg, 7–11.5 kg, and 5–9 kg respectively for underweight, normal weight, overweight, and obese women.

Weight measurements between 8–18 weeks gestation were used to evaluate GWG during early pregnancy. GWG during early pregnancy was evaluated as the average GWG per week up to 18 weeks of pregnancy, and classified as class I (<200 g/week), class II (200–400 g/week), class III (400–600 g/week), and class IV (> 600 g/week) [11].

Gestational BMI gain was categorized as minimal (<5 kg/m^2^), moderate (5–10 kg/m^2^), and excessive (> 10 kg/m^2^) based on evidence from a previous study [7]. Each one point increase in BMI is roughly equivalent to 2.5 kg in weight gain, using the Chinese national average for female height at reproductive age (158 cm, 54 kg) [14].

### Outcome Assessment

Gestational hypertension and preeclampsia were defined according to the International Society for the Study of Hypertension [16]. Gestational hypertension was defined as having a maternal systolic blood pressure (SBP) > 140 mm Hg and/or diastolic blood pressure (DBP) > 90 mmHg, measured on 2 occasions separated by at least 6 hours beginning after 20 weeks gestation. Preeclampsia was defined using the same criteria in conjunction with proteinuria > 300mg on a 24-hour urine collection or proteinuria of at least 1+ on dipstick testing [16].

### Statistical Analysis

Unconditional logistic regression was conducted to calculate odds ratios (ORs) and 95% confidence intervals (CIs) evaluating the association of each factor (pre-pregnancy BMI, total pregnancy GWG, GWG in early pregnancy, and gestational BMI gain) and risk of HDP overall, as well as risk of gestational hypertension and preeclampsia separately. Models were adjusted for other previously identified risk factors for HDP including maternal age at delivery, education level, parity, offspring sex, and gestational week. Separate models were run to evaluate the associations with HDP for total GWG, GWG during early pregnancy, and gestational BMI gain, and all of these models were also adjusted for pre-pregnancy BMI. Additionally, models evaluating the risk of HDP for pre-pregnancy BMI and gestational BMI gain were mutually adjusted. Analyses were further stratified by maternal pre-pregnancy BMI categories, and the interactions between GWG and pre-pregnancy BMI were assessed using a Wald test by including the relevant cross-product terms in the regression models. Linear trends were tested using the Wald test. Statistical analyses were conducted using SAS, version 9.3, (SAS Institute, Inc., Cary, North Carolina) and P < 0.05 was considered statistically significant.

## Results

Table 1 shows selected characteristics of women in the cohort. 1,973 out of 84,656 (2.33%) women were diagnosed with HDP (including 1,244 cases of GH and 729 cases of PE). Women aged over 30 years, nulliparous women, and women who were overweight/ obese before pregnancy were more likely to develop HDP. The mean total GWG among women who developed HDP was 19.52±8.21 kg, higher than that of normotensive women (17.44±7.00 kg). The average GWG up to 18 weeks was also higher among women with HDP (0.219±0.209kg/wk) compared with normotensive women (0.201±0.188kg/wk).

**Table 1:** Distribution of selected characteristics of the cohort stratified by HDP status.

Maternal characteristic	Normotensive (n = 82,683)		HDP(n = 1,973)	
	n/mean	%/SD	n/mean	%/SD
***Age at delivery***				
<25	15664	18.94	237	12.01
25–29	42705	51.65	926	46.93
30–34	19009	22.99	556	28.18
≥35	5305	6.42	254	12.87
***Education Level***				
Less than high school	9515	11.51	222	11.25
High school	37264	45.07	856	43.39
College	31801	38.46	795	40.29
Advanced Degree	4103	4.96	100	5.07
***Parity***				
Nulliparous	68775	83.18	1687	85.50
Multiparous	13908	16.82	286	14.50
***Offspring Sex***				
Male	44097	53.33	1024	51.90
Female	38586	46.67	949	48.10
***Gestational week***				
<37	3030	3.66	266	13.48
37–41	78612	95.08	1691	85.71
≥42	1041	1.26	16	0.81
***Pre-pregnancy BMI(kg/m*** ^***2***^ ***)***				
Under weight (<18.5)	14146	17.11	208	10.54
Normal (18.5–23.9)	63271	76.52	1419	71.92
Overweight (24–27.9)	4648	5.62	273	13.84
Obese(≥28)	618	0.75	73	3.70
***Total GWG(Kg)***	17.44	7.00	19.52	8.21
***Average GWG up to 18 weeks (Kg/wk)*** *	0.201	0.188	0.219	0.209

*Includes 63,603 subjects with at least one weight measurement during the early pregnancy period

Table 2 presents the associations of pre-pregnancy BMI, gestational BMI gain, total GWG, and GWG during early pregnancy in relation to risk of HDP. In the model adjusted for confounders, pre-pregnancy BMI, gestational BMI gain, and GWG were all positively associated with the risk of HDP. Women who were obese prior to pregnancy were about 5 times more likely to develop HDP, compared with women who had a normal pre-pregnancy BMI (adjusted OR = 5.53, 95% CI = 4.28–7.13). A significantly increased risk of HDP was also observed for women with a BMI gain greater than 10 kg/m^2^ during pregnancy (adjusted OR = 3.35, 95% CI = 2.89–3.89). Furthermore, women with GWG above the IOM recommendation had an adjusted OR of 1.72 (95% CI = 1.54–1.93) for developing HDP compared with women who had GWG within the recommendation.

**Table 2:** Associations of pre-pregnancy BMI, gestational BMI gain, total GWG, and GWG during early pregnancy with risk of HDP^a^.

Exposure Variables	Normotensive(n)	HDP(n)	Crude OR(95% CI)	Adjusted OR(95% CI)*
***Pre-pregnancy BMI(kg/m*** ^***2***^ ***)*** ^b^				
Under weight (<18.5)	14146	208	0.66(0.57–0.76)	0.64(0.55–0.74)
Normal (18.5–23.9)	63271	1419	1.00 (ref)	1.00 (ref)
Overweight (24–27.9)	4648	273	2.62(2.29–2.99)	2.66(2.32–3.05)
Obese(≥28)	618	73	5.27(4.11–6.75)	5.53(4.28–7.13)
***Gestational BMI gain (kg/m*** ^***2***^ ***)*** ^b^				
<5	20345	384	1.00 (ref)	1.00 (ref)
5–10	53423	1195	1.19(1.06–1.33)	1.46(1.29–1.64)
≥10	8915	394	2.34(2.03–2.70)	3.35(2.89–3.89)
***Total GWG By IOM Recommendation*** ^b^				
Below	14012	220	0.88(0.75–1.04)	0.88(0.75–1.04)
Within	24927	443	1.00 (ref)	1.00 (ref)
Above	43744	1310	1.69(1.51–1.88)	1.72(1.54–1.93)
***Average GWG up to 18weeks(g/wk)*** ^c^				
<200	37851	803	1.00 (ref)	1.00 (ref)
200–399	15181	342	1.06(0.93–1.21)	1.07(0.94–1.22)
400–599	6199	170	1.29(1.09–1.53)	1.26(1.07–1.50)
≥600	2963	94	1.50(1.20–1.86)	1.48(1.19–1.84)
*P* for trend				<0.01

^a^. Gestational BMI gain, total GWG, and GWG during early pregnancy were evaluated in separate models.

^b^. Adjusted for age at delivery, education level, parity, offspring sex, and gestational week. Additionally, pre-pregnancy BMI and gestational BMI gain were mutually adjusted. Total GWG also adjusted for pre-pregnancy BMI. (n = 84,656)

^c^. Adjusted for age at delivery, education level, parity, offspring sex, and pre-pregnancy BMI. (n = 63,603)

An increasing risk of HDP was also observed as GWG increased during early pregnancy (p trend < 0.01). Compared with women who gained less than 200 grams per week before 18 weeks of pregnancy, the risk of developing HDP was significantly higher among women who gained greater than 400 grams per week (OR = 1.26, 95% CI = 1.07–1.50). Notably, women who gained greater than 600 grams per week during early pregnancy had the highest risk of HDP with an adjusted OR of 1.48 (CI = 1.19–1.84).

Results for gestational BMI gain, total GWG, and GWG during early pregnancy stratified by pre-pregnancy BMI are presented in Table 3. Women with higher BMI gain and GWG during the whole pregnancy had a significantly elevated risk of developing HDP across all pre-pregnancy BMI categories. However, the odds ratio for higher BMI gain and GWG decreased as the pre-pregnancy BMI increased. In particular, women who were underweight before pregnancy and who had higher gestational BMI gain (≥10 kg/m^2^) had the highest risk of developing HDP (adjusted OR = 3.46, CI = 2.07–5.78), whereas the corresponding ORs were still elevated but not as strong in women with higher BMI gain who were overweight or obese before pregnancy (P for heterogeneity < 0.01).

**Table 3:** Associations of gestational BMI gain, total GWG, and GWG during early pregnancy with risk of HDP stratified by pre-pregnancy BMI^a^.

Exposure Variables	under-weight (<18.5 kg/m^2^)		Normal (18.5–23.9 kg/m^2^)		Overweight/Obese(≥24 kg/m^2^)		*P for heterogeneity*
	Crude OR(95% CI)	Adjusted OR(95% CI)	Crude OR(95% CI)	Adjusted OR(95% CI)	Crude OR(95% CI)	Adjusted OR(95% CI)	
***Gestational BMI gain (kg/m*** ^***2***^ ***)*** ^b^							
<5	1.00 (ref)	1.00 (ref)	1.00 (ref)	1.00 (ref)	1.00 (ref)	1.00 (ref)	<0.01
5–10	1.19(0.75–1.87)	1.35(0.85–2.14)	1.35(1.17–1.56)	1.45(1.25–1.67)	1.41(1.11–1.79)	1.47(1.16–1.87)	
≥10	2.80(1.69–4.62)	3.46(2.07–5.78)	2.94(2.49–3.48)	3.40(2.87–4.04)	2.12(1.3–3.45)	2.27(1.39–3.73)	
***Total GWG By IOM Recommendation*** ^b^							
Below	1.31(0.77–2.23)	1.20(0.70–2.05)	0.82(0.68–0.99)	0.83(0.69–1.00)	0.77(0.46–1.28)	0.86(0.52–1.45)	0.35
Within	1.00 (ref)	1.00 (ref)	1.00 (ref)	1.00 (ref)	1.00 (ref)	1.00 (ref)	
Above	2.34(1.69–3.23)	2.72(1.95–3.79)	1.46(1.29–1.66)	1.65(1.45–1.87)	1.45(1.08–1.95)	1.57(1.16–2.11)	
***Average GWG up to 18weeks(g/wk)*** ^c^							
<200	1.00 (ref)	1.00 (ref)	1.00 (ref)	1.00 (ref)	1.00 (ref)	1.00 (ref)	0.71
200–399	0.95(0.63–1.44)	0.94(0.62–1.43)	1.06(0.92–1.24)	1.04(0.90–1.21)	1.27(0.93–1.73)	1.28(0.94–1.74)	
400–599	1.41(0.88–2.28)	1.40(0.87–2.25)	1.25(1.02–1.53)	1.21(0.99–1.49)	1.35(0.92–1.98)	1.34(0.91–1.97)	
≥600	1.38(0.73–2.62)	1.34(0.71–2.55)	1.48(1.14–1.93)	1.42(1.09–1.85)	1.75(1.07–2.86)	1.79(1.09–2.94)	
*P* for trend		0.19		<0.01		<0.01	

^a^. Gestational BMI gain, total GWG, and GWG during early pregnancy were evaluated in separate models.

^b^. Adjusted for age at delivery, education level, parity, offspring sex, and gestational week. (n = 84,656)

^c^. Adjusted for age at delivery, education level, parity, and offspring sex. (n = 63,603)

A different trend was apparent for the association of early pregnancy GWG and HDP stratified by pre-pregnancy BMI (Table 3). No significantly increased risk of HDP was observed among women who were underweight before pregnancy and who had the highest GWG during early pregnancy (highest level vs lowest level: adjusted OR = 1.34, 95% CI = 0.71–2.55). Among women with a pre-pregnancy BMI category classified as normal or overweight/obese, the highest level of GWG (≥600g/week) during early pregnancy significantly elevated the risk of developing HDP, compared to the lowest level of early GWG (among normal weight women: adjusted OR = 1.42, 95% CI = 1.09–1.85; among overweight/obese women: adjusted OR = 1.79, 95% CI = 1.09–2.94). There was, however, no significant heterogeneity between pre-pregnancy BMI categories for the association of GWG during early pregnancy and HDP risk (P for heterogeneity = 0.71).

Additionally, we assessed the associations of pre-pregnancy BMI, gestational BMI gain, total GWG, and GWG during early pregnancy with risk of gestational hypertension and preeclampsia separately. These two outcomes were positively associated with all of the evaluated exposures and the magnitudes of the associations were similar (S1 and S2 Tables).

## Discussion

During the past decades, HDP remains a leading cause of maternal death worldwide [12]. Previous studies conducted in developed countries have indicated that obesity and excessive weight gain during pregnancy pose a significant risk for developing HDP [11, 17, 18]. However, there have been limited epidemiological studies about risk factors for HDP among Chinese women.

In this large cohort study conducted among Chinese women, we found that maternal overweight /obesity before pregnancy was independently associated with an increased risk of HDP, compared with women with lower pre-pregnancy BMI, which is consistent with previous studies [7, 19, 20]. It has been postulated that maternal–fetal immune maladaptation is involved in the pathogenesis of preeclampsia [1, 21, 22]. Adipose tissue is known to be associated with metabolic syndrome including adiposity and hyperlipidemia, which can cause inflammatory changes and then lead to an increase in oxidative stress. This may result in endothelial dysfunction, maternal organ hypo perfusion, and eventually clinical diseases such as HDP [23, 24]. Previous studies have also suggested that adipose tissue may stimulate angiogenesis, which has been recently implicated to be involved in the development of hypertension [25].

Although several previous studies have reported a positive association between excessive GWG and the risk of HDP, the association has not been confirmed due to the generally limited number of studies and the potential limitations of previous study designs [26]. The majority of studies only evaluated the absolute GWG over the entire pregnancy with the risk of HDP and did not distinguish the weight gain driven by edema and the weight gain caused by adiposity. As edema is one of the hallmarks of preeclampsia, the etiologic association of GWG and preeclampsia is uncertain in those studies [7, 27], as weight gain during pregnancy may be the result of edema caused by preeclampsia. To our knowledge, there has been only one study to date, which was conducted in the United Kingdom [11], that attempted to account for the weight caused by edema by assessing weight gain during early pregnancy. That study found a positive association between GWG in early pregnancy and risk of HDP. However, there have been no such studies among Asian women. In the present study, we found that excessive GWG during the whole pregnancy was significantly associated with an increased risk of HDP after adjusting for gestational weeks, which is consistent with previous studies [7, 11]. As BMI is considered by some to be a better measure of adiposity than weight alone [7], we also classified antenatal weight gain according to the net change in BMI, and similarly, excessive BMI gain during pregnancy was shown to be related to an elevated risk of HDP. We additionally evaluated the association of early pregnancy GWG and risk of HDP, and the data indicated that weight gain before 18 weeks gestation was also positively associated with the risk of HDP. This association is unlikely to be explained by edema because edema is unlikely to happen at this stage of pregnancy, which demonstrates that GWG may precede the development of HDP.

It was found in some studies that an increased risk of preeclampsia and gestational hypertension was present in all women with excessive GWG, except those who were underweight prior to pregnancy [9], while others found a positive association across all BMI categories [28]. In order to explore whether the pre-pregnancy BMI may modify the association between pregnancy weight gain and HDP risk, we stratified the association by maternal pre-pregnancy BMI categories, and found a significant association between excessive GWG during the whole pregnancy and an elevated risk of HDP across all the pre-pregnancy BMI categories. Our results also suggested that although the risk of HDP increased with excessive GWG, the risk decreased as pre-pregnancy BMI increased. A similar trend was observed in the stratified results from previous studies [29, 30]. However, the results for early pregnancy GWG with HDP stratified by pre-pregnancy BMI showed a different trend in that the association between early pregnancy GWG and HDP was only significant among women who were normal weight and overweight/obese before pregnancy. The risk of HDP was not significantly increased among women who were underweight and who had higher GWG during early pregnancy, and was highest among women who were overweight/obese before pregnancy and who had the highest level of early GWG. The different trends observed between whole pregnancy GWG and early pregnancy GWG when stratified by pre-pregnancy BMI might be explained by the different levels of edema at the middle or late stage of pregnancy across different pre-pregnancy BMI categories, which needs to be examined by further prospective studies. Excessive weight gain during early pregnancy, which may result from changes in diet and physical activity levels including among women with normal pre-pregnancy BMIs, may lead to an increased risk of HDP through mechanisms involving oxidative stress [7].

Additionally, we evaluated the risk of gestational hypertension and preeclampsia separately, and also observed a positive association with pre-pregnancy BMI category, whole pregnancy GWG, BMI gain, and early pregnancy GWG for both outcomes. The association of excessive GWG during the whole pregnancy and gestational hypertension alone was consistent with a large scale cohort study conducted in the United States [7]. As gestational hypertension is by definition not characterized by proteinuria, women with gestational hypertension would be expected to have less edema; thus, the impact of weight gain should be more likely to be driven by adiposity rather than edema in the process of disease.

Several strengths and limitations should be noted when interpreting the results of our study. A clear strength of this study is the large population-based cohort of pregnant women. Also, the women’s anthropometric characteristics during early pregnancy were available, which allowed us to evaluate the role of both total GWG and early GWG in relation to risk of HDP. To our knowledge, we are only the second study to examine the association of early GWG with the risk of HDP, and the first among Asian women. Several limitations of this study should also be considered. First, though we assessed some potential confounding factors previously reported to influence HDP, there were several other potential confounders that we were not able to evaluate, such as smoking status and family history of HDP, because of the absence of this information in our database. However, we note that the smoking prevalence of women in China is very low [31] and we excluded women with a history of chronic hypertension or cardiovascular disease prior to pregnancy from the study. Additionally, our study relies on a self-reported pre-pregnancy weight, which may be under estimated. Although potential misclassification bias may exist, previous studies suggest that the resulting BMI category from self-reported data rarely alters, and the self-reported weight and height may be considered to be an acceptable substitute for actual measurements [28, 32].

In conclusion, we conducted a large population-based cohort study in China to evaluate the association of pre-pregnancy BMI and GWG with the risk of HDP, and found evidence that pre-pregnancy BMI, total GWG, and early pregnancy GWG were all positively associated with risk of gestational hypertension and preeclampsia. Our results indicate that maternal overweight/obesity, early GWG, and total GWG should be considered in combination in targeting and reducing the risk of HDP. Weight restrictions before and during pregnancy are both important in the control of HDP, but weight gain reduction during pregnancy is much more feasible.

## Supporting Information

S1 Table Associations of pre-pregnancy BMI, gestational BMI gain, and total GWG with risk of subtypes of HDP (n = 84,656). (DOCX)

S2 Table Associations of GWG during early pregnancy (up to 18 weeks) with risk of subtypes of HDP (n = 63,603). (DOCX)
