# Supplementary material for: Pre-Pregnancy BMI, Gestational Weight Gain, and the Risk of Hypertensive Disorders of Pregnancy: A Cohort Study in Wuhan, China
Source: PLoS One. 2015 Aug 25;10(8):e0136291. doi: 10.1371/journal.pone.0136291 (PMC4548954; doi:10.1371/journal.pone.0136291)
Supplement: S1 Table — (DOCX) [file pone.0136291.s001.docx]

**S1 Table.** **Associations of pre-pregnancy BMI, gestational BMI gain, and total GWG with** **risk of subtypes of HDP (n=84,656)**

| Exposure Variables | GH(n=1244) | |  | PE(n=729) | |
| --- | --- | --- | --- | --- | --- |
|  | Crude OR(95% CI) | Adjusted OR(95% CI) |  | Crude OR(95% CI) | Adjusted OR(95% CI) |
| ***Pre-pregnancy BMI(kg/m^2^)*** |  |  |  |  |  |
| Under weight (<18.5) | 0.65(0.54-0.78) | 0.63(0.52-0.76) |  | 0.67(0.53-0.85) | 0.66(0.52-0.84) |
| Normal (18.5–23.9) | 1.00 (ref) | 1.00 (ref) |  | 1.00 (ref) | 1.00 (ref) |
| Overweight (24-27.9) | 2.51(2.12-2.97) | 2.67(2.25-3.17) |  | 2.81(2.27-3.46) | 2.64(2.13-3.28) |
| Obese(≥28) | 5.46(4.04-7.38) | 6.04(4.45-8.19) |  | 4.93(3.28-7.42) | 4.72(3.09-7.21) |
| ***Gestational BMI gain (kg/m^2^)*** |  |  |  |  |  |
| <5 | 1.00 (ref) | 1.00 (ref) |  | 1.00 (ref) | 1.00 (ref) |
| 5-10 | 1.25(1.08-1.45) | 1.45(1.24-1.69) |  | 1.09(0.91-1.31) | 1.47(1.21-1.77) |
| ≥10 | 2.72(2.28-3.26) | 3.49(2.91-4.2) |  | 1.79(1.41-2.27) | 3.07(2.39-3.93) |
| ***Total GWG By IOM Recommendation*** |  |  |  |  |  |
| Below | 0.92(0.75-1.14) | 0.92(0.75-1.14) |  | 0.83(0.64-1.07) | 0.83(0.64-1.08) |
| Within | 1.00 (ref) | 1.00 (ref) |  | 1.00 (ref) | 1.00 (ref) |
| Above | 1.88(1.64-2.17) | 1.78(1.54-2.05) |  | 1.41(1.19-1.68) | 1.64(1.38-1.96) |

*Adjusted for age at delivery, education level, parity, offspring sex, and gestational week. Additionally, pre-pregnancy BMI and gestational BMI gain were mutually adjusted. GWG model was also adjusted for pre-pregnancy BMI.
