# Supplementary material for: Pre-Pregnancy BMI, Gestational Weight Gain, and the Risk of Hypertensive Disorders of Pregnancy: A Cohort Study in Wuhan, China
Source: PLoS One. 2015 Aug 25;10(8):e0136291. doi: 10.1371/journal.pone.0136291 (PMC4548954; doi:10.1371/journal.pone.0136291)
Supplement: S2 Table — (DOCX) [file pone.0136291.s002.docx]

**S2 Table. Associations of GWG during early pregnancy (up to 18 weeks) with risk of subtypes of HDP (n=63,603)**

| Exposure Variables | GH(n=915) | |  | PE(n=494) | |
| --- | --- | --- | --- | --- | --- |
|  | Crude OR(95% CI) | Adjusted OR(95% CI) |  | Crude OR(95% CI) | Adjusted OR(95% CI) |
| Average GWG up to 18weeks(g/wk) |  |  |  |  |  |
| <200 | 1.00 (ref) | 1.00 (ref) |  | 1.00 (ref) | 1.00 (ref) |
| 200-399 | 1.13(0.96-1.32) | 1.14(0.99-1.31) |  | 0.95(0.76-1.18) | 0.89(0.74-1.08) |
| 400-599 | 1.34(1.09-1.65) | 1.23(1.03-1.48) |  | 1.20(0.91-1.60) | 0.97(0.76-1.25) |
| ≥600 | 1.39(1.05-1.84) | 1.43(1.20-1.71) |  | 1.68(1.20-2.36) | 1.69(1.37-2.07) |
| *P* for trend |  | <0.01 |  |  | 0.02 |

*Adjusted for age at delivery, education level, parity, offspring sex and pre-pregnancy BMI.
